# Are All Paraphyllia the Same?

**DOI:** 10.3389/fpls.2020.00858

**Published:** 2020-06-19

**Authors:** Ulyana N. Spirina, Tatiana V. Voronkova, Michael S. Ignatov

**Affiliations:** ^1^Faculty of Biology, Tver State University, Tver, Russia; ^2^Tsitsin Main Botanical Garden, Russian Academy of Sciences, Moscow, Russia; ^3^Faculty of Biology, Lomonosov Moscow State University, Moscow, Russia

**Keywords:** paraphyllia, *Leskea*, abscisic acid, morphogenesis, pseudoparaphyllia, branch primordia, fluridone

## Abstract

Moss paraphyllia, the trichome-like or foliose structures on moss stem surfaces, are usually treated as epidermal outgrowths. However, in some taxa of the moss families Leskeaceae, Neckeraceae, and Amblystegiaceae their distribution along the stem is consistently correlated with parts of the stem surface near branch primordia. In other moss families, Climaciaceae, Hylocomiaceae, and Pseudoleskeaceae the specific paraphyllia-generating epidermal layer produces paraphyllia evenly all along the stem. These results suggest that there are at least two different types of regulation of paraphyllia development; however, both of them may be involved in the morphogenesis of paraphyllia in some families, for example in the Thuidiaceae. Exogenous abscisic acid treatment consistently increases the number of paraphyllia of the *Leskea*-type, and it also induces the development of proximal branch leaves that normally do not develop a lamina above the stem surface. This fact supports conclusions regarding the homology of the *Leskea*-type of paraphyllia with leaves.

## Introduction

Mosses are small plants, and their morphology remains incompletely understood despite a long history of studies. Among the most controversially interpreted features are foliose and filamentose appendages on the stem surface in pleurocarpous mosses, commonly called paraphyllia and pseudoparaphyllia. An interest in their structure has been recently revived after molecular phylogenetic studies showed that many morphological characters used in “classical taxonomy” are enormously homoplasious and can no longer be used as key diagnostic characters. The classification of pleurocarpous mosses at family level is especially flawed, as sporophyte characters have been shown to be extremely plastic during the transition to epiphytism (Huttunen et al., 2004; Huttunen et al., 2012; Hedenäs, 2012). Conversely, previously neglected features of branch primordia, and specifically the arrangement of foliose structures around branch initials, appear stable enough to serve as diagnostic characters for family-level classification (Ignatov, 1999). The terminology of these structures and interpretation of their homology, however, remain incongruent, as different manuals sometimes refer to the same cases in conflicting ways.

**Figure 1:**
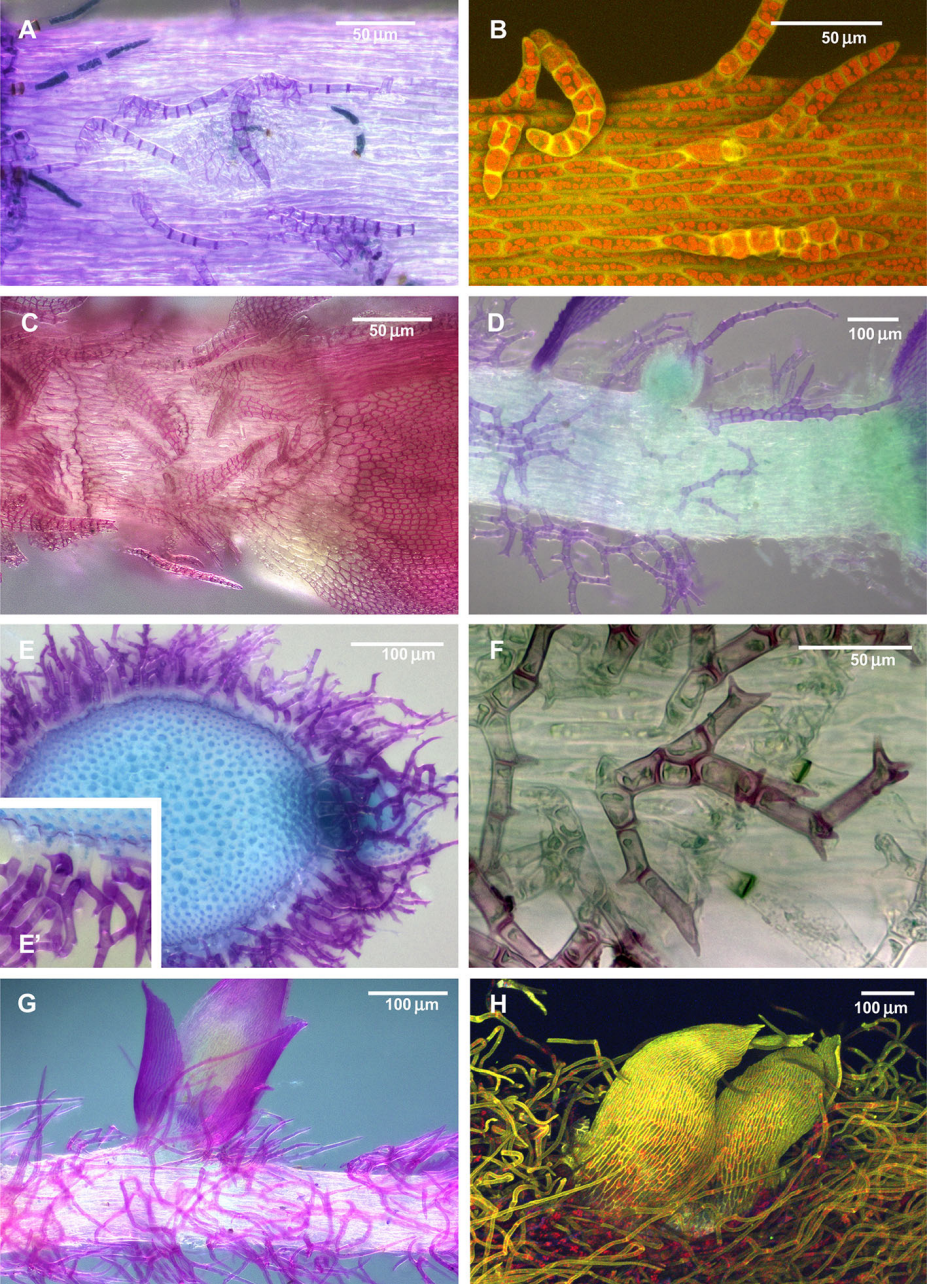
Diversity of paraphyllia in mosses: scattered over stems **(A–C)** or appearing on the majority of cells **(E–H)** or numerous on the stem and more scattered on branches **(D)**. **(A, B)** *Leskea polycarpa*; **(C)** *Cratoneuron filicinum*; **(D)** *Thuidium tamariscinum*, secondary stem with primordium of a tertiary branch; **(E, F)** *Thuidium recognitum*, stem cross-section (E and E', close up of E) and surface view **(F)**; **(G)** *Hylocomium splendens* (Hedw.) Schimp., part of the stem with branch primordium; **(H)** *Climacium dendroides* (Hedw.) F. Weber & D. Mohr, part of the stem with branch primordium. **(A, C–G)**: LM; **(B, H)**: LSCM.

Paraphyllia are filamentose or narrowly lanceolate, rarely ovate, multicellular, branched or unbranched structures, densely or sparsely covering the stems or solitary (**Figure 1**). They were described and illustrated originally by Hedwig (1801), who called them “stupae” (tow), while the authors of “Bryologia Europaea” introduced the name “paraphyllia” (Bruch et al., 1836–1855), a term widely used up to now. Later it was observed that some foliose structures on the stem occur only in proximity to branch primordia, and Warnstorf (1904–1906) introduced another term, “pseudoparaphyllia,” exemplified by the foliose structures around the branch primordia in *Rhynchostegium* (Brachytheciaceae) and *Rhytidiadelphus* (Hylocomiaceae). Ironically, the first world-wide comprehensive overview of pseudoparaphyllia by Ireland (1971) interpreted these two genera as lacking pseudoparaphyllia. Several attempts were made to define the distinction between paraphyllia, pseudoparaphyllia, and proximal branch leaves, and the best-known overviews of structures around branch primordia are those by Akiyama (1990a; 1990b) and Akiyama and Nishimura (1993). These authors suggested that pseudoparaphyllia should be differentiated from proximal branch leaves, for which they proposed the terms “scale-like leaves” or “scaly leaves”. Paraphyllia were treated by these authors as adventive structures (appendages) of the stem epidermis, in contrast to foliose or filamentose structures around branch primordia referred to as pseudoparaphyllia.

Paraphyllia have attracted less attention, and the most detailed overview remains a publication by Bonnot (1967), who discussed possible homologies of paraphyllia, which were treated as appendages of the stem epidermis, additional leaf-like structures, reduced leaves, trichomes, and modified protonematal structures, but always without relation to the branch primordia. Ignatov and Hedenäs (2007) challenged the latter statement showing that in *Alsia, Cratoneuron*, *Leptodon*, and *Leskea*, “paraphyllia” are more abundant near branch primordia or situated, often in pairs, at the locations of undeveloped branch primordia. These authors concluded that in the studied taxa “paraphyllia” may be assumed to be pseudoparaphyllia that have escaped for a certain distance from the branch primordia. However, this suggestion did not meet with wide acceptance.

**Figure 2:**
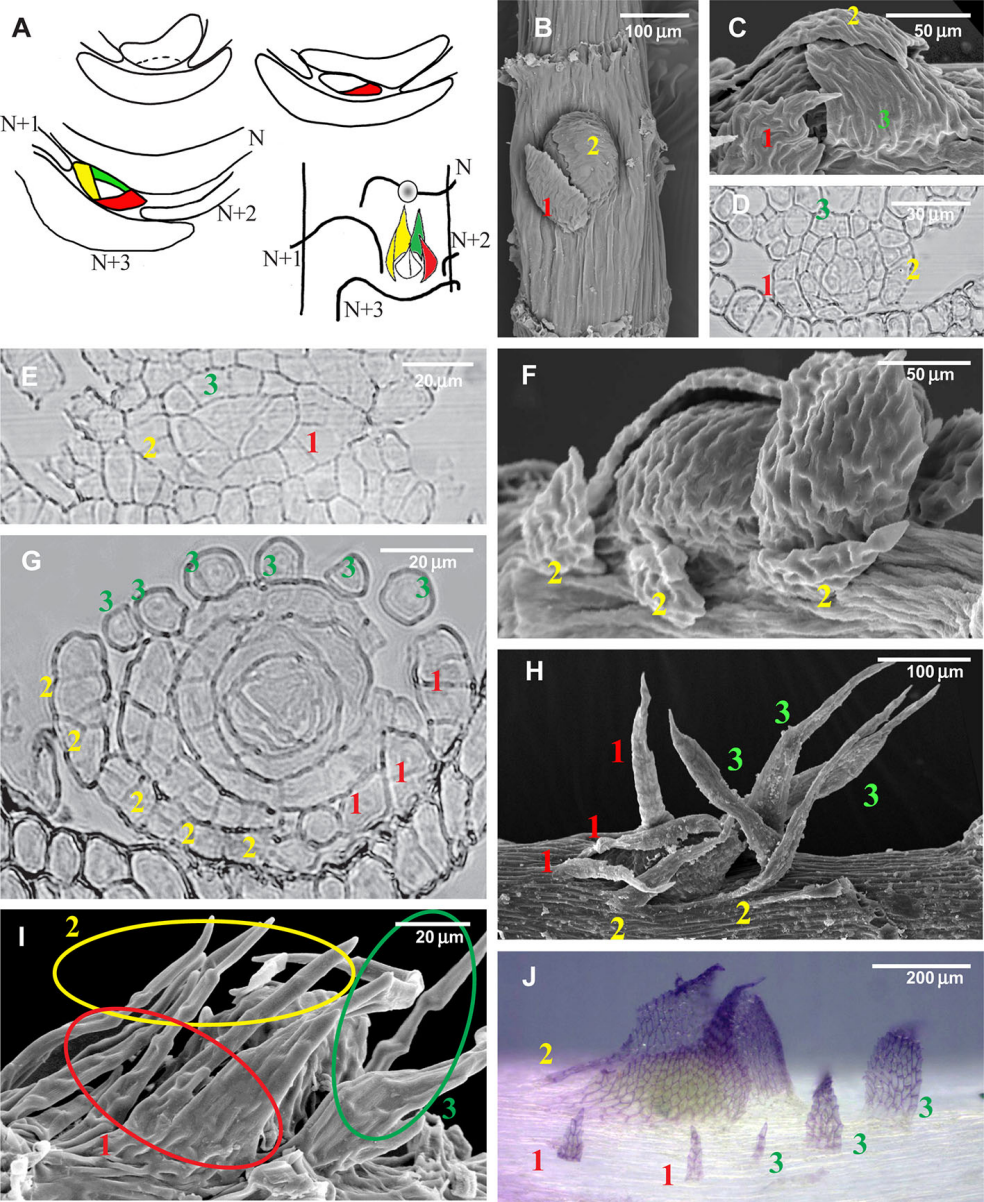
Development of the branch primordium in mosses, illustrating variants of the foliose structures around it. **(A)** Scheme of branch primordium differentiation from leaf N and subsequent displacement towards the axil of leaf N+3 along the path between the decurrences of leaves N+1 and N+2; **(B–D)** Variants of the most common arrangement in pleurocarpous mosses of the proximal branch leaves in *Orthostichella hexasticha* (Schwägr.) W.R. Buck **(B)**, *Campylium stellatum* (Hedw.) C. Jens. **(C)** and *Amblystegium serpens* (Hedw.) Schimp. **(D)**, with the outermost leaf in “four o'clock position” [with the possible switching of left and right spirals, the “four o'clock position” may appear as the “eight o'clock position”, which is still called hereafter the “four o'clock position”]; **(E, G, I)** Development of the branch primordium in *Hypnum cupressiforme*, from the initial, showing formation of merophytes from the trifacial apical cell **(E)**, then the development of leaves strongly incised from the beginning **(G)**, and finally a mature “dormant bud” surrounded by three compound leaves, each of which were traditionally called pseudoparaphyllia **(I)**; **(F, H)** Examples of compound broader proximal branch leaves in *Thamnobryum alopecurum* (Hedw.) Nieuwl. ex Gangulee **(F)** and *Alleniella complanata* (Hedw.) S. Olsson, Enroth & D. Quandt **(H)**, recognized as such due to the apparent phyllotaxis of the branch leaves; **(J)** Branch primordium of *Cratoneuron filicinum*, with first branch leaves compound and remote from the bud. **(B, C, F, H, I)**: SEM; **(J)**: LM, basic fuchsine, and picroindigocarmine.

Later it was shown that moss leaves are not always entire. Proximal branch leaves can be deeply incised, or subdivided to the base, and their parts sometimes occur spaced from each other for a certain distance, appearing to be independent structures, which have been called “compound leaves” (Spirina and Ignatov, 2008; Ignatov and Spirina, 2012; Spirina and Ignatov, 2015). **Figure 2** illustrates the variation in the structure of proximal branch leaves (the outermost leaves around the branch primordium). Most pleurocarps in their development follow the scheme shown in **Figure 2A**, with the first proximal branch leaf appearing in the “four o'clock position” (**Figures 2B–D**). A different situation occurs in some groups, e.g. *Hypnum cupressiforme* Hedw. (**Figures 2E, G, I**), where the first three branch merophytes produce leaves divided into filiform segments which look like independent structures, originating, however, from a single cell. Furthermore, the parts of such compound leaves can be somewhat displaced (**Figures 2F, H**), and ultimately stand apart from the branch initial (**Figure 2J**). The cases where the parts of compound branch leaves appear distant from the branch primordia raise questions about the distinction between paraphyllia and compound proximal branch leaves. The homology of the previously mentioned structures thus requires better definition, and this is in the main focus of the present paper. Specifically, the hypothesis that we test is whether foliose and filamentose structures on the surface of moss stems, excluding rhizoids, axillary hairs, and gemmae, can be classified in two or more groups based on their structure and distribution along the stem.

In addition to morphological studies we conducted experiments with exogenous abscisic acid (ABA). The selection of ABA is based on preliminary studies with three phytohormones, cytokinin, auxin, and ABA, on the growth of *Leskea polycarpa* Hedw. Only ABA showed an apparent effect on morphology, specifically the conspicuous increase in the number of paraphyllia after exogenous ABA application, and therefore we undertook more detailed studies.

ABA is an important compound in plants, involved in the regulation of plant growth, germination, stomatal movement, responses to abiotic stresses, as well as the stresses caused by viruses and bacteria (Zhang, 2014; Alazem and Lin, 2017; Alazem et al., 2017). Although it is widely accepted that ABA regulates stresses (Osakabe et al., 2014; Yoshida et al., 2014; Shi et al., 2015), there are still few reports that show a direct role of ABA.

Cho et al. (2009) review ABA studies in *Physcomitrella*, confirming the similarity of its physiological responses to that in seed plants. For the morphogenetic role of ABA, however, Cho et al. (2009) noted only membrane fragility after freezing, which is increased in untreated protonematal cells. There are also reports of the thickening of cell walls (Tintelnot, 2006), induction of brood cells (Goode et al., 1993), induction of tmema cells (Decker et al., 2006) and inhibition of cytokinin-induced bud formation on protonema (Christianson, 2000).

The putative importance of ABA studies to the paraphyllia/pseudoparaphyllia problem can be related to its ability to promote seed dormancy in angiosperms (Nee et al., 2016; Shu et al., 2016; Shu et al., 2017; Shuai et al., 2017) and to mediate plasmodesmata closure in the bud meristems of *Populus*, thereby inducing bud dormancy (Tylewicz et al., 2018). Kitagawa et al. (2019) demonstrated the regulation of plasmodesmata permeability in the moss *Physcomitrella patens* (Hedw.) Bruch & Schimp. and showed that the molecular trafficking through them is rapid and reversibly restricted by abscisic acid. Thus a kind of release from dormancy may be better understood using deeper ABA studies. Branch primordia in the majority of pleurocarpous moss genera have a specific variant of dormancy: branch primordia start to develop, produce small-sized foliose protective structures covering the bud, and then revert to a dormant state but are promptly released from it in the case of damage to a stem apical cell. Although ABA was never considered for dormancy in mosses, its effect on structures associated with dormancy seems to merit being tested.

## Materials And Methods

### Morphological Studies

#### Species Sampling

*Leskea polycarpa* was the main focus of the present study as its paraphyllia are the easiest to count. This is a widespread epiphytic moss, common on tree trunks in the temperate zone of the Northern Hemisphere. Its habitats periodically experience drought lasting from several days to several weeks. Intensive growth of *Leskea polycarpa* in the Moscow region starts in the second half of spring. As the species was the main subject of our ABA investigation, we undertook anatomical studies by sectioning to elucidate the sub-surface structure of its branch primordia.

Two other mosses studied for paraphyllia distribution along the stem and also for the effect of ABA were chosen from genera of different families: *Leptodon* (Neckeraceae) and *Cratoneuron* (Amblystegiaceae).

Genera from the following families known to have paraphyllia were re-studied with light microscopy to observe the distribution pattern of the paraphyllia: Amblystegiaceae (*Cratoneuron*, *Palustriella*), Climaciaceae (*Climacium, Pleuroziopsis*), Leskeaceae (*Leskea*), Neckeraceae (*Leptodon*, *Neckera* p.p.), Pseudoleskeaceae (*Lescuraea* s.l., and probably related to it *Rhytidiopsis*), Theliaceae (*Thelia*), Thuidiaceae (*Abietinella*, *Actinothuidium*, *Boulaya*, *Bryochenea*, *Bryonoguchia*, *Haplocladium*, *Hylocomiopsis*, *Helodium*, *Pelekium*, *Rauiella*, *Thuidium*).

We include illustrations of some examples of other taxa from previous studies, and some additional images prepared for better illustration of the structure of the paraphyllia and proximal branch leaves.

A list of taxa and studied specimens is available in **Supplementary Materials 1**.

#### Distribution of Paraphyllia Along the Stem

**Figure 3:**
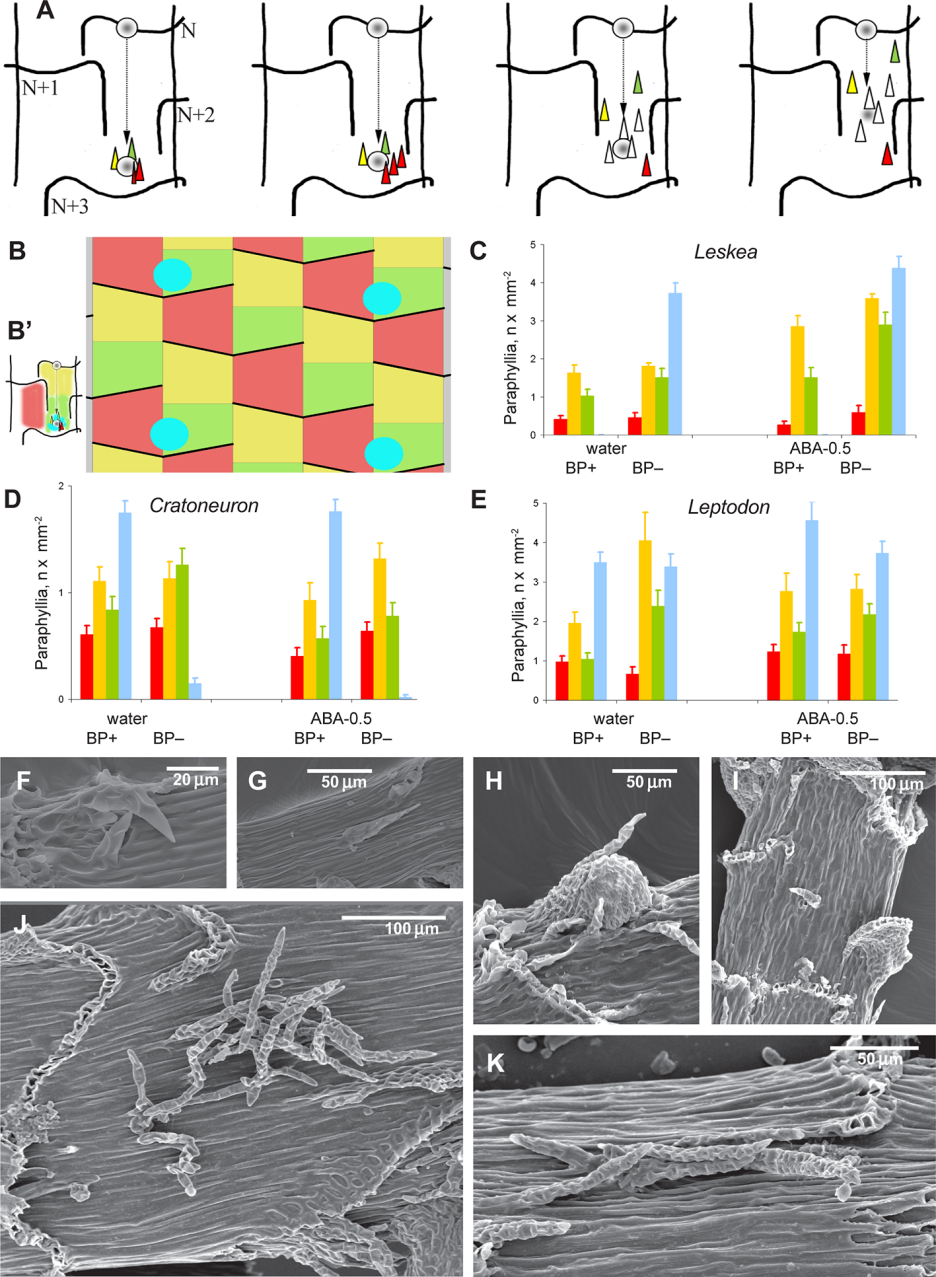
Distribution of paraphyllia of the *Leskea*-type along the stem. **(A)** The scheme, based on observations of the branch primordium and paraphyllia in *Leskea*-type positions (cf. examples in **(F–K)**; the scheme illustrates the most common position of these paraphyllia along the path of branch primordium displacement from the “mother-leaf “, N, to the axil on the N+3 leaf (cf. also **Figure 2A**); **(B)** Scheme of whole stem surface circumference for a moss with 2/5 phyllotaxis, showing zones used for paraphyllia counts (B' helps to link these zones to the scheme in **A**) colors denote: blue is the area immediately close to the branch primordium; green is the lower half of the “internode”, up to the upper level of the leaf N+2, yellow is the area above the green zone up to the leaf N, and red is the part of the stem below leaf N on the side other than where the branch primordium originated; note that leaf decurrences in the scheme are not shown for simplicity; **(C–E)**. The number of paraphyllia in experiments with three species, *Leskea polycarpa* **(C)**, *Cratoneuron filicinum* **(D)**, *Leptodon smithii* **(E)**, for the three zones of the stem as defined in the scheme in “B” and the area near branch primordia (blue); the count was performed separately for “internodes” with branch primordia (BP+) and without them (BP–); some of the plants were studied after ABA experiments, and their paraphyllia were counted separately, showing an especially large increase of paraphyllia in the yellow zone. See more data and statistics in **Supplementary Materials 2**. **(F–K)** examples of the position of foliose structures between decurrences of leaves N+1 and N+2 (cf. **Figures 2A** and **3B**), in *Hygroamblystegium varium* **(F)**: branch primordium, **(G)**: paraphyllium, *Leskea polycarpa* **(H)**: branch primordium, **(I)**: paraphyllium, *Neckera californica* **(J)** and *Leptodon smithii* **(K)**. **(F–K)**: SEM.

Observations of the distribution of the paraphyllia along the stem were made in *Leskea polycarpa, Leptodon smithii* D. Mohr, and *Cratoneuron filicinum* (Sull.) Spruce. The area of the stem was subdivided into three zones (**Figures 3A, B**), based on the observation that paraphyllia of *Leskea*-type occur on the stem mainly along the route of the branch primordium from “mother-leaf” N to a destination in the axil of the leaf N+3. The area between the decurrencies of leaves N+1 and N+2 has paraphyllia more often than the rest.

All leaves were removed from the shoots with thin tweezers, stems were stained with fuchsine, and photographed under a light microscope Olympus-CX41 with digital camera Infinity 2-2. Paraphyllia were counted from the images, consulting the original glass slide in uncertain cases. Stems on a glass slide were covered by another slide, allowing for observations of both sides of the stem. Plants from herbaria were studied in the same way.

#### Anatomical Studies

Anatomical studies were conducted with *Leskea polycarpa* to characterize the structure of the branch primordia. To prepare anatomical sections, the upper parts of shoots (with leaves removed) were fixed in a 2.5% glutaraldehyde solution for 7 days, post-fixed in a 1% OsO_4_ water solution for 3 hours, washed in water. After washing, the material was dehydrated in an alcohol series (20%, 40%, 60%, 80% and 96% alcohol), alcohol–acetone mixture (1:1), and acetone for 1 hour in each solution, soaked in an acetone–resin mixture series (3:1, 1:1, 1:3) for 12, 24 and 3 hours respectively, and embedded in epon-araldite resin as recommended by the manufacturer. The resin was polymerized at 60°C for 24 hours. Serial longitudinal, transverse, and oblique sections were cut 2 µm thick with glass knives, placed on glass slides without mounting medium, stained with 0.01% berberine or its combination with DAPI and scanned under a laser scanning confocal microscope Olympus FV-1000 based on Olympus BX61, using or combining 405 and 473 nm lasers. Z-stacks of several scans were usually obtained and are presented here.

Two series of longitudinal sections and three series of transverse ones were made, and some interesting cases observed under the microscope were studied.

#### Supplementary Studies

The structure of branch primordia was studied for pleurocarpous species of several families, mainly for taxonomic purposes. In the present paper we illustrate the least known cases involving the complete reduction of proximal branch leaves, and their division to the base, causing them to appear to be compound.

These studies involved light microscopy (LM), scanning electron microscopy (SEM), and laser scanning confocal microscopy (LSCM).

**LM** analysis: paraphyllia were described with regard to their shape and structure, position, and arrangement on the stem, contrast being enhanced by fuchsine and picroindigocarmine staining.

For **SEM** analysis the shoots were fixed in a 4% glutaraldehyde solution for 7 days, incubated in a 1% OsO_4_ water solution for 6 hours, washed in water, dehydrated through an ascending alcohol–acetone series, dried at the critical point, and coated with gold. Prepared shoots were observed and photographed under a LEO430 scanning electron microscope (Carl Zeiss, Germany). For some shoots SEM observations of gold-coated samples were performed with the SEM Jeol 6380, without additional preparation.

Living material was observed and photographed with **LSCM** with similar staining by berberine or berberine/DAPI, without fixation. Combinations of lasers 405, 473, and 576 were used for maximally detailed pictures. Scan series (mostly at 1024×1024 pixels, 20–70 scans) are presented here as Z-stacked images, or, in some cases, photo-galleries of parts of series.

### ABA Experiments

#### Sampling

Observations were conducted on *Leskea polycarpa, Cratoneuron filicinum. Leptodon smithii*, and *Thuidium tamariscinum* (Hedw.) Schimp., representatives of four different families. The selection depended on the position and number of paraphyllia which are readily countable. *Thuidium tamariscinum* has very dense and numerous paraphyllia on stem and primary branches, but on secondary branches their number is comparable with that in *Leskea*; thus only secondary branches were studied for this species. Species with very dense and interwoven paraphyllia, e.g., *Hylocomium* and *Climacium* were not studied, because it is almost impossible to count paraphyllia on their stems and branches, and paraphyllia are situated on all cells.

#### Cultivation

*Leskea polycarpa* was placed in Petri dishes (diameter 9 cm) as ten tufts of ca. 2×2 cm, without separating plants into individuals. They were put on one layer of filter paper moistened with 5 ml of distilled water or an aqueous solution of ABA (Sigma, Germany) at a concentration of 0.1 μmol, 0.04 μmol, 0.01 μmol, 0.002 μmol, hereafter referred to according to the amount applied in μmol as ABA-0.5, ABA-0,2, ABA-0.05, and ABA-0.01. Petri dishes were placed in a Sanyo Environmental Test Chamber MLR-352H: temperature + 7°С/+ 12°С (night/day), light period 10 hours, PPFD - 14 μmolm^-2^s^-1^ for 21 days.

#### Field Experiment

In addition to the experiments in the test chamber, we explored the effect of ABA on *Leskea in vivo* to exclude the possibility that the observed effect on paraphyllia depends on cultivation in the permanently humid condition of the Petri dish. ABA was applied directly to tufts of *Leskea polycarpa* growing in the park of the Tsitsin Main Botanical Garden in Moscow, on four trunks: two of *Acer tataricum* L., and one each of *A. platanoides* L. and *Malus sylvestris* Mill., at 1.5 m above the ground. Two tufts 5 x 5 cm of *Leskea* were moistened on each trunk: one with ABA-0.5 solution and one with distilled water. A soft painting brush was used for wetting moss turfs. All turfs were situated on the same eastern side of the trunk in similar conditions. The experiment was conducted in the period following 25 April 2017, i.e. when most epiphytic mosses had resumed growth in Moscow (in the rainy and warmer days after winter).

#### Counting and Statistics

After 21 days of cultivation, 50 shoots from each Petri dish were selected. All leaves were removed from the stems with thin tweezers. The stems were stained with fuchsine, placed on a glass slide, and covered by another glass slide, allowing observations from both sides of the stem. The stems were examined under the light microscope (Olympus-CX41) and photographed with a digital camera (Infinity 2-2). Stem diameter was measured, and the number of paraphyllia was counted from the images. The number of paraphyllia was calculated for one square millimeter of the stem surface. Cultivated plants, plants from the field, and herbarium materials were studied in the same way.

The significance of the ABA effect was evaluated by the T-test in MS Excel (**Supplementary Materials 2**).

#### Fluridone Test

To make sure that it is the presence of ABA that causes changes in the morphogenesis of paraphyllia, we used a test with fluridone, an ABA biogenesis inhibitor (Popova, 1995; Klíčová et al., 2002; Velini et al., 2010; Shu et al., 2017) that may reduce the content of the free form of ABA by up to 40% (Stetsenko et al., 2015). Fluridone (Sigma, Germany) at a concentration of 15 μmol was applied in 1 ml quantities to *Leskea* cultivated with ABA and without it. The first test, which consisted of adding fluridone only once at the beginning of the cultivation period, showed inconsistent results. The absence of effect could be explained by the decomposition of fluridone as direct light destroys its molecules over a single day (Müller and Applebyki, 2011). Therefore a second test was performed with the application of fluridone three times a week (altogether nine times for 21 days). The significance of the impact of fluridone was evaluated by an ANOVA test in PAST (Hammer et al., 2001).

#### Brachytheciaceae Test

Brachytheciaceae is known as a family in which the branch primordia lack leaves formed from the first and second branch merophytes, which therefore appear underdeveloped, forming no foliose structures above the stem surface (Ignatov, 1999; Spirina and Ignatov, 2005). We have used ABA to check if such “hidden” leaves can develop a lamina above the stem surface. The experiment was conducted with the same protocol as in *Leskea* in the test chamber. Plants of *Brachythecium rutabulum* (Hedw.) Schimp. which were used for this study, were collected from the park in the Tsitsin Botanical Garden in Moscow.

## Results

### Morphological Studies

#### Distribution of Paraphyllia Along the Stem

The evaluation of the distribution of paraphyllia along the stem was performed quantitatively using zones associated with the morphogenesis of branch primordia (**Figures 3A, B**). The number and distribution of paraphyllia in *Leptodon smithii*, *Cratoneuron filicinum*, and *Leskea polycarpa*, species from three different families, show approximately the same pattern. Paraphyllia are most abundant near the branch primordium and along the route that the primordium passes in its ontogenetic history on the path from mother leaf N (cf. also **Figures 2A** and **3A**) to the axil of the leaf N+3. The lowest number of paraphyllia occurs in the “red area” (**Figures 3C–E**), i.e. the area below a half of the leaf that cut off the cell which is later developed into the internode with the branch primordium (**Figures 2A** and **3A**).

Visual evaluation of a wide selection of taxa with paraphyllia confirms that the pattern seen in *Leskea, Cratoneuron*, and *Leptodon* is also characteristic for *Neckera menziesii* Drumm.*, N. californica* Hook. & Arn., *Palustriella commutata* (Hedw.) Ochyra*, P. decipiens* (De Not.) Ochyra*, Leskea gracilescens* Hedw.*, L. obtusa* Renauld & Cardot, and *Hygroamblystegium varium* (Hedw.) Mönk. (**Figures 3F–K**): either numerous or scattered paraphyllia occur in the “green” and “yellow” zones as they are delimited in **Figure 3B**.

**Figure 4:**
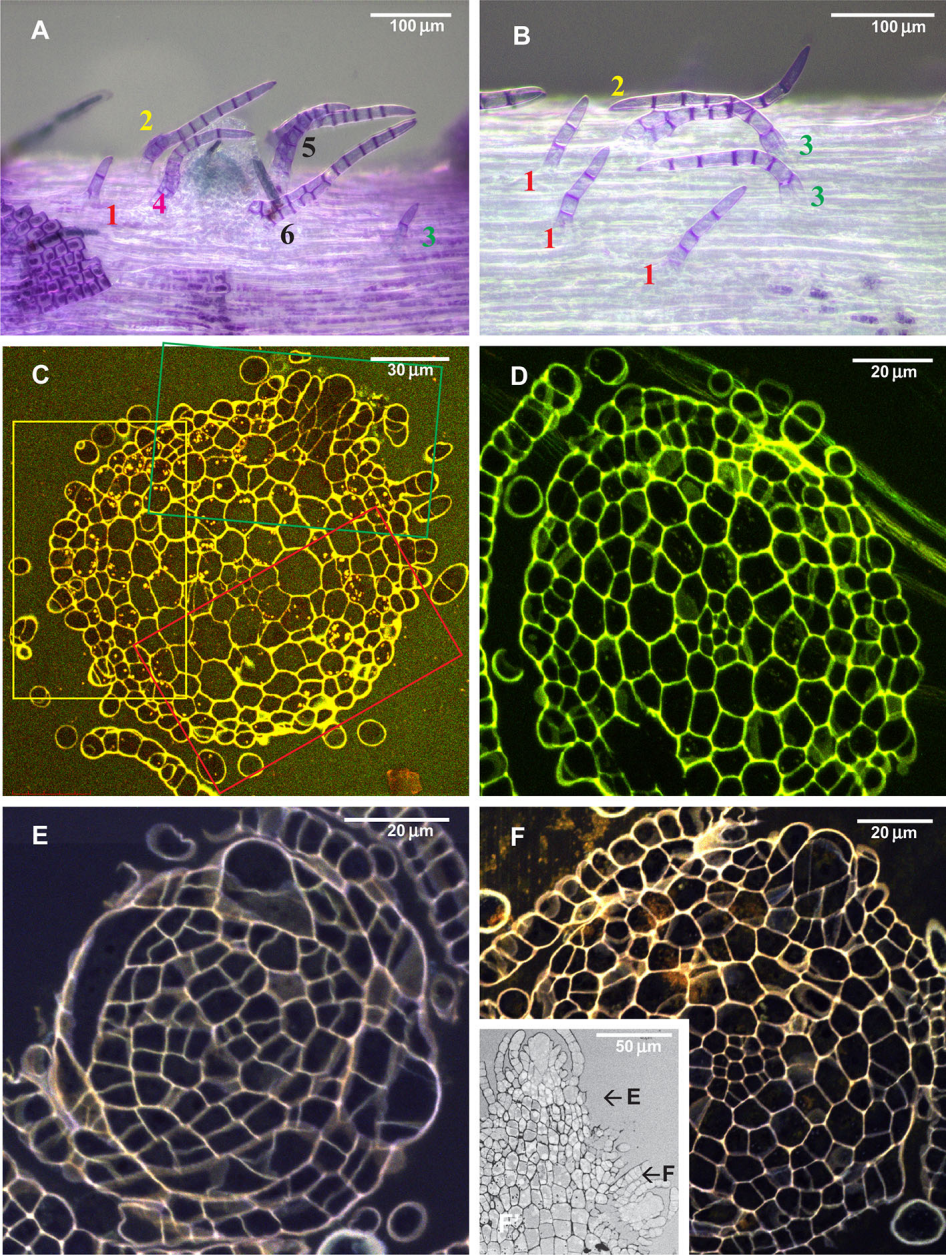
Arrangement of paraphyllia around developed and undeveloped branch primordia of *Leskea polycarpa*, as seen on the stem **(A, B)** and in stem transverse sections **(C, D)**, and the early stages of branch primordium development **(E, F)**. **(A)** Compound proximal branch leaves (“escaped pseudoparaphyllia”) around branch primordium (for numbering cf. **Figure 2**); **(B)** Paraphyllia arranged with apparent phyllotaxis around a vestigial primordium; **(C, D)** Transverse section across the “apices” of the primordium and vestigial primordium shown in “A” and “B”; **(E, F)** Transverse section of *Leskea* shoot at 46 and 78 µm from the apex, showing that the branch initials have a very broad base, which probably causes the branch itself to be rather indefinitely delimited from the surrounding cells, differentiated from the same cell at an early stage of development shown in **Figure 2A**. **(A, B)**: LM, basic fuchsine, and picroindigocarmine.

Within the proximity to branch primordia (the “green” zone in **Figure 3D**) the arrangement of paraphyllia around the branch primordia often has a more or less clear phyllotaxis. Therefore the branch merophyte numbers to which paraphyllia belong can be interpreted (**Figures 2J** and **4A**) by comparison with classical schemes of stem morphogenesis (Berthier, 1971), partly reproduced in **Figures 2A** and **3A**. The further from branch primordia, the less regular they are, grading to an irregular arrangement, or even occasionally forming short longitudinal rows.

#### Anatomical Studies

Paraphyllia or compound proximal branch leaves in *Leskea polycarpa* are more numerous around branch primordia (**Figure 4A**); however, in some cases paraphyllia retain a definite phyllotaxis around a point which has no indications of a branch initial on the surface (**Figure 4B**). Transverse cross-sections made at this particular location allowed us to observe only one slightly enlarged subepidermal cell (**Figure 4D**), quite unlike the most common appearance of a branch primordium shown in **Figure 4C**. The concentration of paraphyllia around the branch primordia can also be seen in transverse sections (rectangles in **Figure 4C**). The transverse section of *Leskea* shoot close to the apex (**Figures 4E, F**) shows that the cells forming the “internode” and the branch initials (cf. **Figure 2A**) have a very broad base, which probably causes the apical and subapical branch cells to be rather indefinitely delimited from the surrounding cells. Foliose structures around the branch initial cells are only moderately tightly appressed to the apical cell and later the whole space between young stem leaves is filled by foliose structures around the branch initial cell (**Figures 4F–F**).

#### Paraphyllia of Different Types

**Figure 5:**
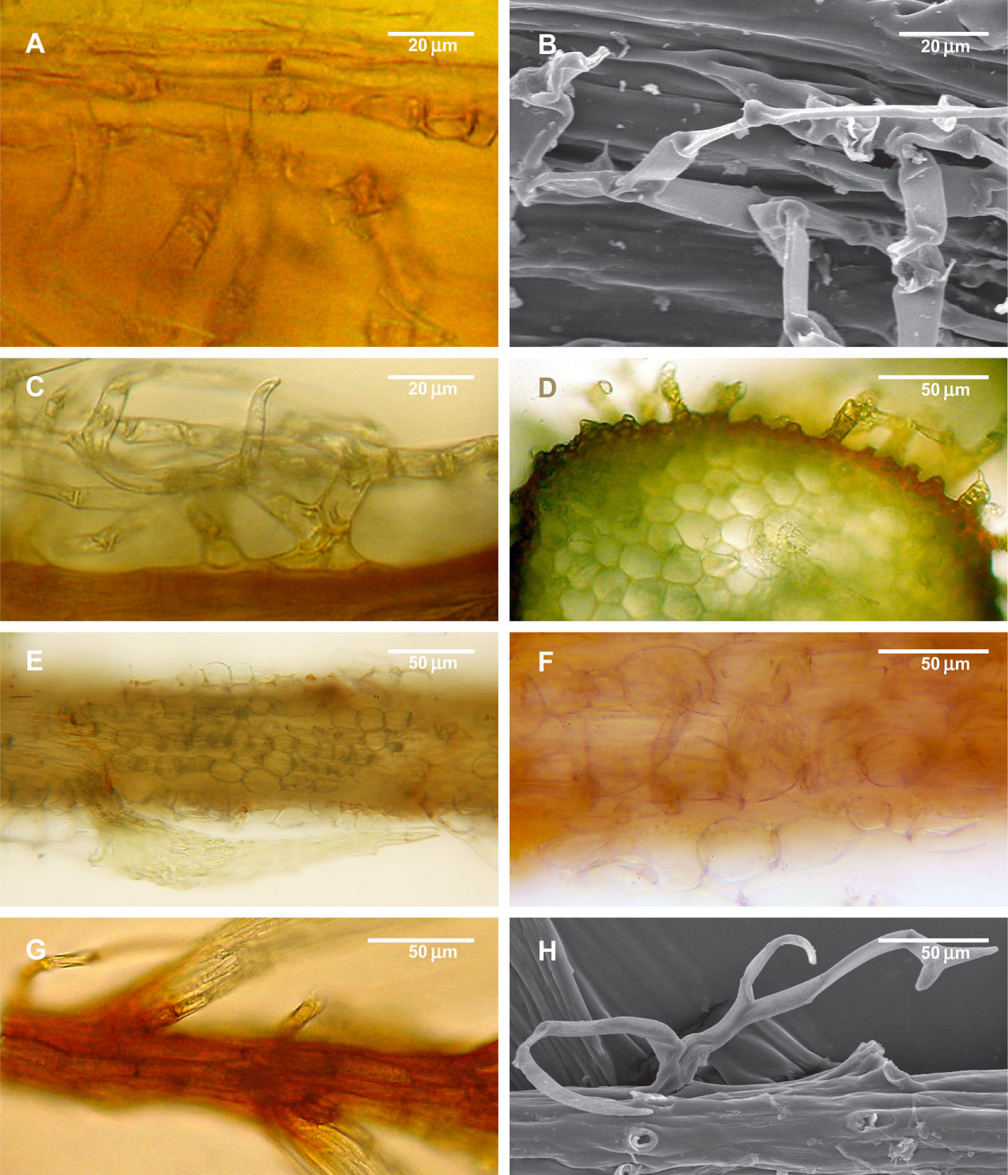
Structure of paraphyllia of the *Climacium*-type. **(A–D)** *Climacium dendroides*, showing paraphyllia in moderately inflated longitudinal ridges; **(E–F)** *Pleuroziopsis ruthenica* (Weinm.) Kindb. ex E. Britton, longitudinal ridges of inflated cells, very rarely producing also paraphyllia; **(G, H)** *Hylocomium splendens*, branch apical part with a row of more or less inflated cells; stem surface with most of the paraphyllia removed. **(A, C–G)**: LM; **(B, H)**: SEM.

Unlike the above-mentioned genera where paraphyllia are concentrated around the branch primordia, there are moss genera in which the paraphyllia are dense, situated on almost all the cells of the stem surface, or if scattered then still evenly distributed along the stem, and usually more or less arranged in longitudinal rows. This pattern is characteristic for *Climacium* (Climaciaceae), *Hylocomiastrum*, *Hylocomium*, *Loeskeobryum*, and *Rhytidiopsis* (Hylocomiaceae) (**Figure 5**). *Pleuroziopsis*, another genus of Climaciaceae, usually has rows of unusually inflated cells, with sparse paraphyllia.

Species of the Pseudoleskeaceae also have paraphyllia in rows, but they do not cover the stem completely; sometimes they are only scattered, and there are species in the family that lack paraphyllia completely.

Species of the Thuidiaceae have stems often densely covered by paraphyllia, which are occasionally arranged in conspicuous rows (e.g. in *Bryonoguchia*), although sometimes they are very homogeneous (e.g. in *Bryochenaea, Pelekium*). At the same time, their branches are less abundantly paraphyllose, and the concentration of paraphyllia near the branch bases is obvious (**Figure 1D**), and their arrangement is similar to that in *Leskea*. We interpret these paraphyllia as being largely of the *Climacium*-type, but in thinner branches their development is regulated in the same way as in *Leskea*.

### ABA Experiments

#### ABA Experiments *In Vitro*

**Figure 6:**
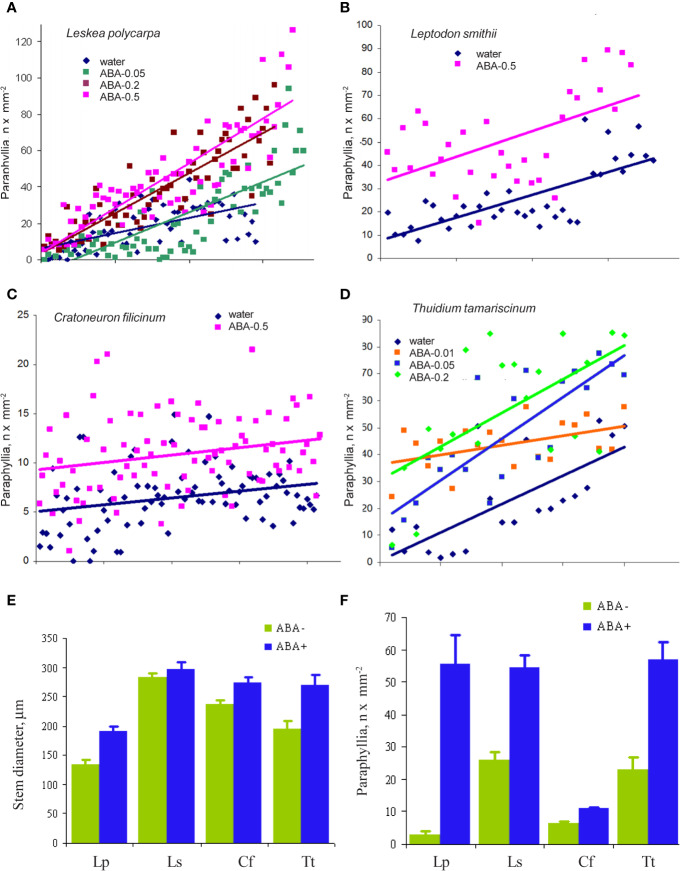
The effect of exogenous ABA on the formation of paraphyllia in four moss species after four weeks of growth in Petri dishes with aqueous solutions of ABA. Axis Y: number of paraphyllia per mm^2^. Counts of paraphyllia number per square unit are arranged along the X axis by the following method: studied stems were numbered and then sorted within each series (i.e. ABA-0.5, ABA-0.2, etc) by stem diameter, from narrowest to widest, thus the position on X axis is the position number of a given stem in its series. Such distributions allow us to compare clouds of dots in different series. **(A)** *Leskea polycarpa*; **(B)** *Leptodon smithii*; **(C)** *Cratoneuron filicinum*; **(D)** *Thuidium tamariscinum*. Color corresponds to ABA concentration; **(E)** Stem diameter from experiments shown in “A–D”; **(F)** Number of paraphyllia from experiments shown in **(A–D)**. See also **Supplementary Materials 3**.

The cultivation of *Leskea polycarpa, Cratoneuron filicinum, Leptodon smithii*, and *Thuidium tamariscinum* with ABA solutions of different concentrations *in vitro* led to an increase in the number of paraphyllia on the tips of growing shoots during the treatment period (**Figure 6**). This increase was significant for all four studied species.

In the first three species paraphyllia were counted on the stem, while in the tripinnate branched plants of *Thuidium tamariscinum* secondary branches were used, as on the stem and primary branches paraphyllia are too numerous to count.

#### ABA Experiments *In Vivo*

Since *Leskea polycarpa* occurs on many trees within the park area of the Tsitsin Main Botanical Garden in Moscow, we also conducted a “field experiment”, moistening tufts of *Leskea* on the trunk of maple with ABA solution at the time of its growing season in spring, using the same series of concentrations as *in vitro* experiments in the test chamber. The results obtained show the same response as in cultivation experiments (**Supplementary Materials 3**).

#### Fluridone Test on *Leskea*

**Figure 7:**
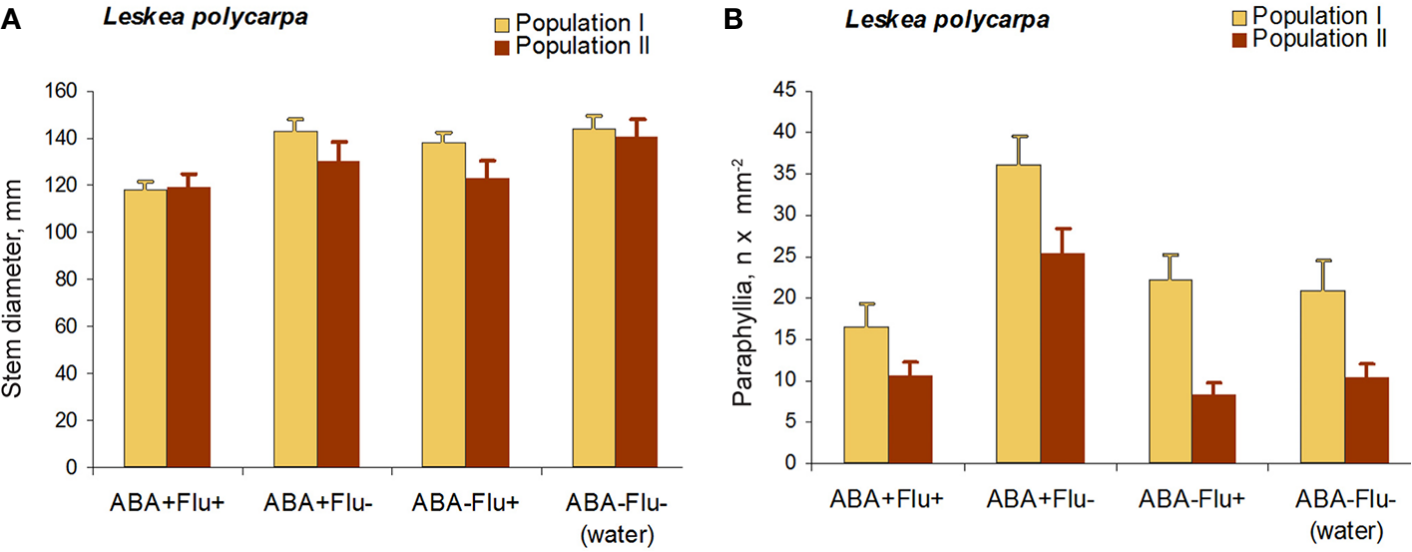
Fluridone inhibition of the ABA effect on the growth of paraphyllia in *Leskea polycarpa*. Experiments were conducted with plants from two populations, the first with somewhat larger plants and more numerous paraphyllia. **(A)** stem width, changing slightly after growth with ABA and indistinctly narrowed in the series with fluridone; **(B)** Number of paraphyllia per square μm, showing their considerable increase after ABA treatment, which is, however, inhibited by fluridone. See also **Supplementary Materials 4**.

The application of fluridone (FLU) inhibited the increase in the numbers of paraphyllia by exogenous ABA (**Figure 7**). The reduction in paraphyllia numbers in the variant [ABA+ FLU+] compared with [ABA+ FLU–] was 63 and 51% in two series of experiments with plants from two populations (**Figure 7**), and was statistically significant supported by an ANOVA test (**Supplementary Materials 4**), p < 0.001. The effect on the stem diameter was less apparent, although significant at p < 0.05 (**Supplementary Materials 4**).

#### *Brachythecium* Test

**Figure 8:**
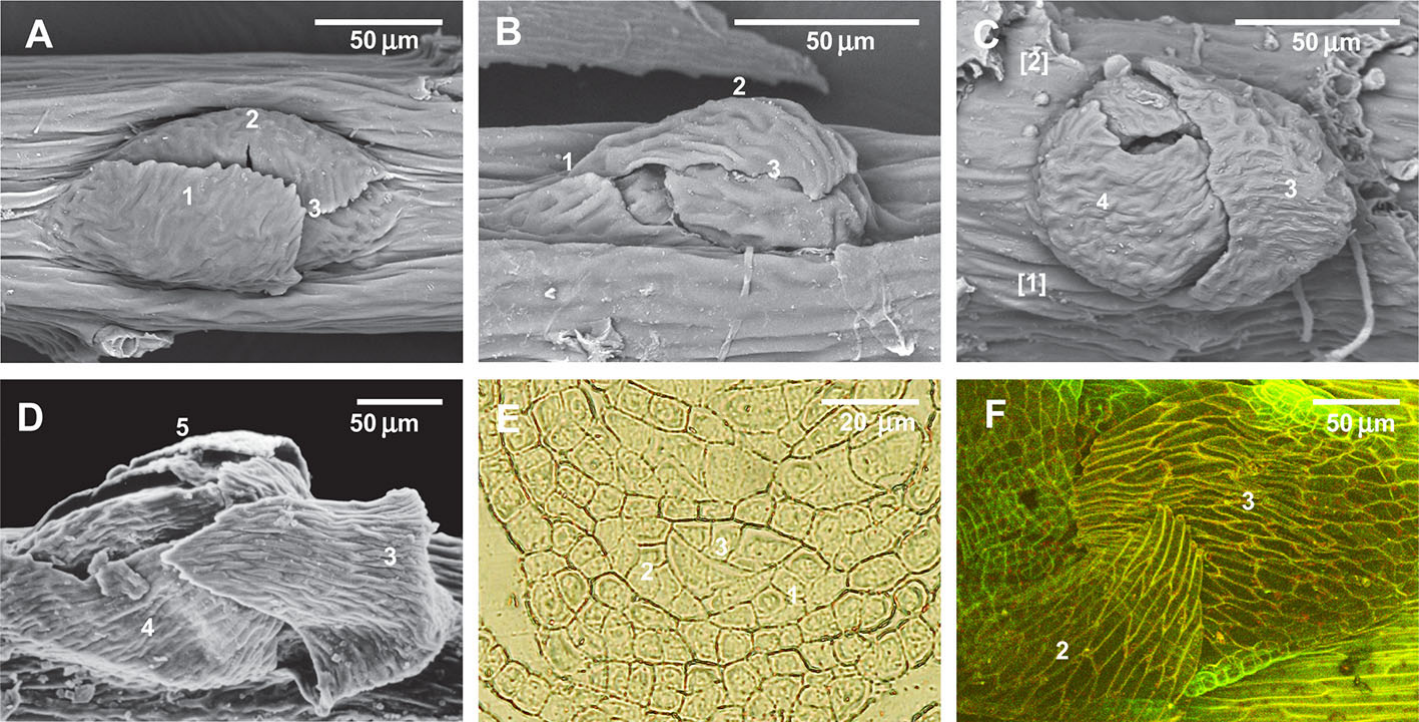
Illustration of the reduction of the first proximal branch leaves in Lembophyllaceae s.l. (incl. Orthostichellaceae), the common absence of the first and second proximal branch leaves in Brachytheciaceae and their occasional re-appearance in response to ABA treatment, and the indifference to ABA of paraphyllia of Climaciaceae and Hylocomiaceae. **(A)** *Orthostichella hexasticha*, with a normally developed first proximal branch leaf; **(B)** *Pilotrichella cuspidata* Broth. with strongly reduced first proximal branch leaf; **(C)** *Camptochaete angustata* (Mitt.) Reichardt with totally reduced first and second proximal branch leaves; **(D)** *Brachythecium glareosum* (Bruch ex Spruce) Schimp., dormant bud, typical for the Brachytheciaceae family, with the outermost leaf in the “twelve o'clock position” due to complete reduction of the first and second proximal branch leaves; **(E)** *Brachythecium rivulare* Schimp., transverse section of branch primordium, showing the very early stage, confirming that the first merophytes are normally differentiated, but later do not form leaves; **(F)** *Brachythecium rutabulum* from cultivation with ABA, with developed second (or first)? proximal branch leaf in the “four o'clock position”. **(A–D)**: SEM; **(F)**: LSCM.

Since previous experiments found that ABA induces the development of leaf-like structures on the stem in places where they are commonly absent, we undertook another test with mosses that are plastic in branch primordium development. **Figures 8A–C** show a parallel situation in the family Lembophyllaceae s.l., where there is great variation in branch primordium structure, allowing stages of reduction to be illustrated. By contrast, in Brachytheciaceae the branch primordium structure is conserved in terms of proximal branch leaf arrangement, so that the outermost leaf is always pointed downwards (**Figure 8D**). Spirina and Ignatov (2005) showed, however, that at the early developmental stage all three leaves exist (**Figure 8E**, cf. **Figure 2A**); therefore the outermost existing leaf (**Figure 8D**) is morphologically the third in sequence. Our attempt to reinstate the reduced leaves in *Brachythecium rutabulum* achieved a certain success. We found that five out of 50 studied primordia developed the second proximal branch leaf (**Figure 8F**) in the ABA-0.5 series, and one poorly developed second branch leaf was also noticed in the control series with distilled water. Statistics are difficult to apply in this case, but hundreds of observations on this character in studies for worldwide taxonomic treatments (Ignatov and Huttunen, 2002) and on specimens of Brachytheciaceae for biodiversity studies never revealed any cases where the outermost leaf in any genus of Brachytheciaceae was seen in a lateral position. They were always found to be stable in retaining the “*Brachythecium*-type of branch primordia” as defined by Ignatov (1999); Ignatov and Huttunen (2002), and Ignatov and Hedenäs (2007).

## Discussion

The observations presented here demonstrate that there are at least two different patterns in the morphogenetic regulation of moss paraphyllia. The examples of *Leskea, Cratoneuron*, and *Leptodon* illustrate a pattern centered on the branch primordium, characterized by phyllotaxis in the paraphyllia arrangement and their concentration along the route passed by the bud in its morphogenetic history.

The homology of the *Leskea*-type of paraphyllia as a principally foliose structure probably has to be assumed as a compromise. On the one hand, there is an obvious regulation causing phyllotaxis even around a hardly discernible branch apical cell (**Figures 4A–D**); on the other hand, the paraphyllia appear irregularly arranged in the parts of internodes distant from the branch primordium. Such flexibility in paraphyllia development makes *Leskea* potentially a model object for studies of morphogenetic regulation in mosses. The release of some “normally undeveloped” structures from the “reduced state”, like the second proximal branch leaf in *Brachythecium* (**Figure 8F**), is similar to the response of *Leskea* to ABA. Therefore this could be additional evidence for the homology of *Leskea*-type paraphyllia with leaves. Thus, the general definition of paraphyllia as structures not related to branch primordia is no longer valid. We suggest naming this pattern as “paraphyllia of the *Leskea-*type”, which is characterized by phyllotaxis and a higher concentration of paraphyllia in the “green” and “yellow” zones (**Figure 3B**).

Paraphyllia of another type, the *Climacium*-type, are arranged mostly in longitudinal rows that cover the stem evenly and are not concentrated around branch primordia. Proximal branch leaves in these species are contrastingly different from paraphyllia (**Figure 1**). In some genera, e.g. *Lescuraea*, paraphyllia may be moderately dense to very sparse, but even in the latter case their even distribution along the stem is apparent and the “red zone” (**Figure 3B**) is never devoid of paraphyllia. Norris and Ignatov (2000) suggested that paraphyllia of the *Climacium*-type may be compared with micronemata (“microrhizoids”) in Mniaceae; the differences of micronemata from macronemata (“normal rhizoids”) in this family were described by Koponen (1968). The development of paraphyllia of the *Climacium*-type and micronemata is initiated from longitudinally oriented structures of modified and somewhat inflated epidermal cells: ridges in *Climacium* and *Pleuroziopsis* (**Figure 5**), and micronemata initials in Mniaceae. Micronemata are evenly distributed on stem surfaces; at least, there is no definite zone on the stem where they are fewer, in contrast to paraphyllia of the *Leskea*-type. However, there are important distinctions between micronemata and paraphyllia of the *Climacium*-type: micronemata lack chloroplasts and look like rhizoids, and their cell walls are oblique, while in paraphyllia of the *Climacium*-type most cell walls are transverse; micronemata originate closer to the center of micronemata initials, whereas paraphyllia in *Climacium* and *Hylocomium* are located at the cell end. Thus it is difficult to confirm their homology, if understood traditionally, while admitting that each structure may be homologous to just one organ. Alternatively, we may explain the structure of paraphyllia by the interference of different regulatory networks.

Kofuji and Hasebe (2014) found that the sporophyte generation in mosses has only one type of stem cell, whereas in gametophytes there are seven types: apical cells of chlorenchyma, caulonema, gametophore, leaf, rhizoid, antheridium, and archegonium. It seems that their number is even more than seven, if we consider the still little-known axillary hairs and paraphyses. As initials most of these organs originate from the stem surface, and it is likely that their regulatory systems interfere, thus the structures of “hybrid” identity may be developed, and *Climacium*-type of paraphyllia may be an example of this.

The present study demonstrates the possibility of using pleurocarpous mosses as a promising model object for studies that cannot be conducted with acrocarpous mosses, including *Physcomitrella*, as well as *Syntrichia* and *Ceratodon*. The acrocarpous mosses lack protective structures on the stem around branch initials that are easily observable. Conversely, the species of the order Hypnales, to which most pleurocarps belong, have diverse stem structures that are suitable for microscopic studies, including numerical counting and observation of their spatial distribution. There are still numerous disadvantages for their use as model objects, such as the lack of a reference genome, their representation by only wild types, and their rather slow growth. However, the study of the transition to dormancy and back might be an important issue on which mosses of the order Hypnales might shed light. Their success in modern biota, where they constitute no less than 40% of the diversity of all moss species, exceeding that of any other moss order, probably depends on their capability to postpone the development of young branches. Most Hypnales retain branch primordia in the state of “ready” or “steady”, and immediately start growth after wetting, which is especially significant for epiphytic mosses, of which pleurocarps constitute the vast majority.

## Data Availability Statement

The datasets generated for this study are available on request to the corresponding author.

## Author Contributions

General plan (MI), plant fixation and embedding (US), anatomical study (US), microscopy (US, TV, MI), ABA experiments (TV), manuscript preparation (MI, US, TV).

## Funding

Russian Foundation for Basic Research 19-04-00976 and MBG Institutional research project 18-118021490111-5.

## Conflict of Interest

The authors declare that the research was conducted in the absence of any commercial or financial relationships that could be construed as a potential conflict of interest.
