# Supplementary material for: Are All Paraphyllia the Same?
Source: Front Plant Sci. 2020 Jun 19;11:858. doi: 10.3389/fpls.2020.00858 (PMC7318891; doi:10.3389/fpls.2020.00858)
Supplement: Supplementary file 1 [file Table_1.docx]

Supplementary Material

**Supplementary materials 1: Voucher specimens used for paraphyllia and proximal branch leaf studies**

**1. Voucher specimens from populations used in the complex of studies, including ABA and fluridone experiments**

*Leskea polycarpa* – Russia, Moscow, Ignatov, 17-1002 MHA

*Cratoneuron filicinum* – Russia, Moscow , Ignatov & Ignatova 17-1003 MHA

*Leptodon smithii* – Russia, Caucasus, Ignatov & Ignatova MHA9046973

*Thuidium tamariscinum* – Germany, Ignatov 12 June 2016 s.n. MHA

*Brachythecium rutabulum* – Russia, Moscow, Ignatov, 17-1001 MHA.

**2. Specimens presented in illustrations in the same order as in figures (except mentioned above)**

**Figure 1.**

*Thuidium recognitum* – Russia, Moscow, Ignatov MHA9119644.

*Hylocomium splendens* – Russia, Moscow, Ignatov & Ignatova 17-1004 MHA

*Climacium dendroides* – Russia, Moscow, Ignatov & Ignatova, MHA9012687

**Figure 2.**

*Orthostichella hexasticha* – Cuba, Pócs et al. 9183/A, MHA

*Campylium stellatum –* Russia, Moscow, Ignatov MHA9011591

*Amblystegium serpens* – Russia, Moscow, Spirina 4.X.2008, s.n. MHA

*Hypnum cupressiforme –* Russia, Krasnodar Territory, Ignatov & Ignatova, MHA9037917

*Thamnobryum alopecurum –* Russia, Caucasus, Ignatov & Ignatova MHA9119204

*Alleniella complanata –* Russia, Caucasus, Ignatov & Ignatova MHA9017256.

**Figure 3.**

*Hygroamblystegium varium –* Russia, Volgograd, Ignatov, MHA9036558

*Neckera californica* USA, Ignatov, MW9000862

**Figure 5.**

*Climacium dendroides* – same as for Fig. 1.

*Pleuroziopsis ruthenica* – Russia, Kuril Islands, Ignatov MHA9042294

*Hylocomium splendens* – same as for Fig. 1.

**Figure 8.**

*Orthostichella hexasticha* – same as for Fig. 2.

*Pilotrichella cuspidata* – Tanzania, Pócs 87039/B

*Camptochaete angustata* – New Zealand, Fife &Tan, 94-274, MHA

*Brachythecium glareosum* – Russia, Volgograd, Ignatov, MHA9006421

*Brachythecium rutabulum* – Russia, Tver, Notov July 2005 TVER

**3. Specimens presented only in morphological studies, indicating paraphyllia type**

| Family | Species | Voucher | *Leskea-* type paraphyllia | *Climacium*-type paraphyllia |
| --- | --- | --- | --- | --- |
| Amblystegiaceae | *Cratoneuron filicinum* | Russia, Moscow Province, Grigorove, Ignatov & Ignatova 17-1003 MHA. | + | – |
| Amblystegiaceae | *Palustriella falacata* | Russia, Perm, Bezgodov, MHA9038647 | + | – |
| Climaciaceae | *Climacium dendroides* | Russia, Moscow, Ignatov & Ignatova, MHA9012687 | – | + |
| Climaciaceae | *Climacium japonicus* | Russia, Kuril Islands, Ignatov MHA9012908 | – | + |
| Climaciaceae | *Pleuroziopsis ruthenica* | Russia, Kuril Islands, Ignatov MHA9042294 | – | + |
| Hylocomiaceae | *Hylocomiastrum pyrenaicum* | Russia, Altai, Ignatov & Ignatova MW9044466 | – | + |
| Hylocomiaceae | *Hylocomiastrum umbratum* | Russia, Tver, Ignatov MHA9037079 | – | + |
| Hylocomiaceae | *Hylocomium splendens* | Russia, Moscow, Ignatov & Ignatova 17-1004 MHA | – | + |
| Hylocomiaceae | *Loeskeobryum brevirostrum* | Russia, Caucasus, Akatova, MHA9023124 | – | + |
| Hylocomiaceae | *Loeskeobryum cavifolium* | Japan, Higuchi, MHA9061707 | – | + |
| Hylocomiaceae | *Rhytidiopsis robustus* | USA, Norris 101931 MHA | – | + |
| Leskeaceae | *Leskea polycarpa* | Russia, Moscow, Ignatov, 17-1002 MHA | + | – |
| Neckeraceae | *Leptodon smithii* | Russia, Caucasus, Ignatov & Ignatova 9046973 | + | – |
| Neckeraceae | *Neckera californica* | USA, Ignatov, MW9000862 | + | – |
| Neckeraceae | *Neckera menziesii* | USA, Shevock, MW9075640 | + | – |
| Pseudoleskeaceae | *Lescuraea plicata* | Russia, Caucasus, Ignatov et al., MHA9014430 | – | + |
| Pseudoleskeaceae | *Lescuraea radicosa* | Russia, Perm, Bezgodov, MHA9014437 | – | + |
| Theliaceae | *Thelia asprella* | USA, Ignatov, MHA9058841 | – | + |
| Theliaceae | *Thelia hirtella* | USA, Allen, MHA9058850 | – | + |
| Thuidiaceae | *Abietinella abietina* | Russia, Yakutia, Ivanova MHA9000152 |  |  |
| Thuidiaceae | *Actinothuidium hookeri* subsp. *boreale* | Russia. Khabarovsk Territory, Ignatov MHA9000401 | + | + |
| Thuidiaceae | *Boulaya mittenii* | Russia. Primorsky Territory, Ignatov MW9004382 | – | + |
| Thuidiaceae | *Bryochenea* | Russia. Primorsky Territory, Ignatov MHA9030025 | – | + |
| Thuidiaceae | *Bryonoguchia* | Russia. Primorsky Territory, Ignatov MHA9101464 | – | + |
| Thuidiaceae | *Haplocladium angustifolium* | Russia, Altai, Ignatov & Ignatova 12-842 MHA | – | + |
| Thuidiaceae | *Helodium* | Russia, Moscow Province, Ignatov MHA9035613 | – | + |
| Thuidiaceae | *Hylocomiopsis ovicarpa* | Russia. Primorsky Territory, Ignatov & Ignatova, MHA9037095 | – | + |
| Thuidiaceae | *Pelekium pygmaeum* | Russia. Primorsky Territory, Ignatov & Ignatova MHA9038956 | – | + |
| Thuidiaceae | *Rauiella fujisana* | Russia. Primorsky Territory, Ignatov. MHA9048222 | – | + |
| Thuidiaceae | *Thuidium tamariscinum* | Germany. Ignatov 12 June 2016 s.n. MHA | + | + |
