# Supplementary material for: Are All Paraphyllia the Same?
Source: Front Plant Sci. 2020 Jun 19;11:858. doi: 10.3389/fpls.2020.00858 (PMC7318891; doi:10.3389/fpls.2020.00858)
Supplement: Supplementary file 2 [file Table_2.docx]

Supplementary Material

**Supplementary materials 2: Data and statistics for exogenous ABA treatment effect on paraphyllia in four moss species**

| Processing options | Stem diameter, μm | Number of paraphyllia, per1μm^2^ | Increase in paraphyllia number compared to control,  % | Number of shoots studied in each sample |
| --- | --- | --- | --- | --- |
| *Leskea polycarpa*, in vitro | | | | |
| Control (water) | 143,6 ± 20.6 | 22,0 ± 6.9 | - | 58 |
| ABA-0.05 | 146,3 ± 17.1 | 37,6 ± 4.2 | 71 | 68 |
| ABA-0.2 | 146,6 ± 12.7 | 46,5 ± 8.6 | 112 | 63 |
| ABA-0.5 | 165,7 ± 34.2 | 54,6 ± 9.2 | 148 | 71 |
| *Leskea polycarpa*, in vivo, on the tree | | | | |
| Control (water) * | 234,5 ± 14.1 | 0,5 ± 0.2 | - | 30 |
| ABA-0.01 * | 246,8 ± 15.3 | 0,8 ± 0.3 | 59 | 30 |
| ABA-0.05 * | 253,8 ± 14.3 | 1,4 ± 0.4 | 183 | 30 |
| ABA-0.25 * | 241,8 ± 14.1 | 2,1 ± 0.4 | 324 | 30 |
| Control (water) ** | 106,6 ± 8.2 | 0,7 ± 0.3 | - | 20 |
| ABA-0.25 ** | 149,5 ± 9.7 | 1,8 ± 0.6 | 155 | 20 |
| Control (water) *** | 311,9 ± 25.3 | 2,1 ± 0.5 | - | 20 |
| ABA-0.25 *** | 359,6 ± 21 | 4,3 ± 1.1 | 102 | 20 |
| Control (water)**** | 386,0 ± 36.2 | 6,6 ± 0.8 | - | 20 |
| ABA-0.25 **** | 450,8 ± 32.1 | 14,9 ± 1.3 | 125 | 20 |
| *Thuidium tamariscinum*, in vitro | | | | |
| Control (water) | 210,0 ± 18.4 | 26,5 ± 5.4 | - | 20 |
| ABA-0.01 | 338,9 ± 27.1 | 45,2 ± 4.1 | 71 | 20 |
| ABA-0.05 | 256,5 ± 24.2 | 44,2 ± 6.7 | 67 | 20 |
| ABA-0.25 | 284,0 ± 24.8 | 58,6 ± 6.4 | 121 | 20 |
| *Cratoneuron filicinum*, in vitro | | | | |
| Control (water) | 236,9 ± 17.1 | 6,5 ± 0.8 | - | 85 |
| ABA-0.5 | 274,7 ± 17.2 | 10,9 ± 1.0 | 68 | 85 |
| *Leptodon smithii*, in vitro | | | | |
| Control (water) | 265,9 ± 18.9 | 17,5 ± 4.3 | - | 35 |
| ABA-0.5 | 271,4 ± 26.6 | 43,0 ± 6.2 | 145 | 35 |

* on *Malus sylvestris* Mill., ** on *Acer platanoides* L., *** on *Acer tataricum* L. trunk 1, **** on *Acer tataricum* trunk 2. All are from park area of the Tsitsin Main Botanical Garden, trees scattered, Leskea populations diffusely shaded.

**P-value in T- test**

| *Leskea polycarpa* in vitro | | | |  | |  | *Leskea polycarpa* in vitro | | | |  |
| --- | --- | --- | --- | --- | --- | --- | --- | --- | --- | --- | --- |
| stem diameter, μ | | | |  | |  | paraphyllia per 1mm^2^ | | | |  |
|  | n = 71 | | n = 63 | n = 68 | |  |  | | n = 71 | n = 63 | n = 68 |
|  | ABA-0.05 | | ABA-0.2 | ABA-0.5 | |  |  | | ABA-0.05 | ABA-0.2 | ABA-0.5 |
| ABA-0.2 | 0,0038 | |  |  | |  | ABA-0.2 | | 1,14E-05 |  |  |
| ABA-0.5 | 0,0004 | | 0,4486 |  | |  | ABA-0.5 | | 3,36E-08 | 9,59E-02 |  |
| water | 0,7136 | | 0,0017 | 0,0001 | |  | water | | 2,33E-01 | 2,75E-09 | 1,21E-11 |
| n = 58 |  | |  |  | |  | n = 58 | |  |  |  |
| *Leskea polycarpa* in vitro | | | | | | | |  | |  |  |
| paraphyllia per 1mm^2^ | | | | | | | |  | |  |  |
|  | | ABA-0.05 | | | ABA-0.2 | | | ABA-0.5 | |  |  |
| ABA-0.2 | | 0,0091 | | |  | | |  | |  |  |
| ABA-0.5 | | 0,3939 | | | 1,47E-05 | | |  | |  |  |
| water | | 0,0003 | | | 1,4E-05 | | | 5,899E-06 | |  |  |
| n = 50 | |  | | |  | | |  | |  |  |

| *Leskea polycarpa* in vivo1 | | | | | | |  | | |  | | *Leskea polycarpa* in vivo2 | | | | | | |  |
| --- | --- | --- | --- | --- | --- | --- | --- | --- | --- | --- | --- | --- | --- | --- | --- | --- | --- | --- | --- |
| stem diameter, mμ | | | | | | |  | | |  | | paraphyllia per 1mm^2^ | | | | | | |  |
|  | water | |  | | | |  | | |  | |  | | | | water |  | |  |
| ABA-0.25 | 0,000373 | |  | | | |  | | |  | | ABA-0.25 | | | | 0,042672 |  | |  |
| n = 30 |  | |  | | | |  | | |  | | n = 30 | | | |  |  | |  |
| Leskea polycarpa in vivo3 | | | | | | |  | | |  | | Leskea polycarpa in vivo4 | | | | | | |  |
| stem diameter, mμ | | | | | | |  | | |  | | paraphyllia per 1mm^2^ | | | | | | |  |
|  | water | | |  | | |  | | |  | |  | | | | water | |  |  |
| ABA-0.25 | 0,196921 | | |  | | |  | | |  | | ABA-0.25 | | | | 0,003776 | |  |  |
| n = 20 |  | | |  | | |  | | |  | | n = 20 | | | |  | |  |  |
| *Leptodon smithii* | | | | | | | |  | | |  | | | |  |  |  |  |  |
| stem diameter, μ | | | | |  | paraphyllia per 1mm^2^ | | | | | | | | |  |  |  |  |  |
|  | | water | | |  |  | | | water | | | | |  |  |  |  |  |  |
| ABA-0.5 | | 0,3879 | | |  | ABA-0.5 | | | 4,69E-08 | | | | |  |  |  |  |  |  |
| n = 35 | |  | | |  | n = 35 | |  | | | | |  | |  |  |  |  |  |

| *Cratoneuron filicinum* | | | | | | | | | | | |  | | |  |  |  |  |  |  |
| --- | --- | --- | --- | --- | --- | --- | --- | --- | --- | --- | --- | --- | --- | --- | --- | --- | --- | --- | --- | --- |
| stem diameter, μ | | | |  | paraphyllia per 1mm^2^ | | | | | | | | | |  |  |  |  |  |  |
|  | | water | |  |  | | | water | | | | | |  |  |  |  |  |  |  |
| ABA-0.5 | | 0,0003 | |  | ABA-0.5 | | | 3,20E-13 | | | | | |  |  |  |  |  |  |  |
| n = 85 | |  | |  | n = 85 | | |  | | | | | |  |  |  |  |  |  |  |
| *Thuidium tamariscinum* | | | | | |  | | | |  |  | | | | | |  |  | |  |
| stem diameter, mμ | | | | | |  | | | |  | paraphyllia per 1mm^2^ | | | | | | | | | |
|  | ABA-0,01 | | ABA-0.05 | | | ABA-0.25 |  | |  | | | | ABA-0,01 | | | ABA-0.05 | | | ABA-0.25 | |
| ABA-0.05 | 0,003416 | |  | | |  |  | | ABA-0.05 | | | | 0,521 | | |  | | |  | |
| ABA-0.25 | 0,003049 | | 0,5171 | | |  |  | | ABA-0.25 | | | | 0,0343 | | | 0,1638 | | |  | |
| water | 5,25E-07 | | 0,0178 | | | 0,001 |  | | water | | | | 5E-05 | | | 0,0004 | | | 1,14E-05 | |
| n = 20 |  | |  | | |  |  | | n = 20 | | | |  | | |  | | |  | |
