# Supplementary material for: Are All Paraphyllia the Same?
Source: Front Plant Sci. 2020 Jun 19;11:858. doi: 10.3389/fpls.2020.00858 (PMC7318891; doi:10.3389/fpls.2020.00858)
Supplement: Supplementary file 4 [file Table_4.docx]

Supplementary Material

**Supplementary materials 4: Statistics for Figure 7. Fluridone inhibition of ABA effect on paraphyllia number**

Test for equal means (ANOVA) **for paraphyllia**

(In repeated measures ANOVA)

Tukey's pairwise test

Tukey's Q below the diagonal, p above the diagonal

**Significant comparisons marked bold**

|  | A+F+ | A+F- | A-F+ | A-F-(control) |
| --- | --- | --- | --- | --- |
| A+F+ |  | **0.0002126** | 0.4258 | 0.9646 |
| A+F- | 5.848 |  | **7.792E-06** | **3.061E-05** |
| A-F+ | 2.149 | 7.997 |  | 0.7231 |
| A-F-(k) | 0.6715 | 6.519 | 1.477 |  |

Sum of sqrs df Mean square F p (same)

Between groups: 5422.2 3 1807.4 12.32 **4.133E-07**

Within groups: 18184.5 124 146.649

Total: 23606.7 127

omega2: 0.2097

Levene´s test for homogeneity of variance, from means p (same): 0.0005422

Levene´s test, from medians p (same): 0.001915

Welch F test in the case of unequal variances: F=9.179, df=67.44, p=3.572E-05

Test for equal means **for stem diameter**

no significant difference

Sum of sqrs df Mean square F p (same)

Between groups: 8604.54 3 2868.18 1.91 0.1313 (p>0.05)

Within groups: 186171 124 1501.38

Total: 194775 127

omega2: 0.02089

Levene´s test for homogeneity of variance, from means p (same): 0.2215

Levene´s test, from medians p (same): 0.247

Welch F test in the case of unequal variances: F=2.076, df=68.48, p=0.1114
